# Parameter-Independent Deformation Behaviour of Diagonally Reinforced Doubly Re-Entrant Honeycomb

**DOI:** 10.3390/polym16213082

**Published:** 2024-10-31

**Authors:** Levente Széles, Richárd Horváth, Mihály Réger

**Affiliations:** 1 Doctoral School on Materials Sciences and Technologies, Óbuda University, H-1034 Budapest, Hungary; 2 Bánki Donát Faculty of Mechanical and Safety Engineering, Óbuda University, H-1034 Budapest, Hungary

**Keywords:** lattice design, reinforced lattice, deformation stabilisation, parameter-independent behaviour, FEM, additive manufacturing, hyperelastic material model

## Abstract

In this study, a novel unit cell design is proposed, which eliminates the buckling tendency of the auxetic honeycomb. The novel unit cell design is a more balanced, diagonally reinforced doubly re-entrant auxetic honeycomb structure (x-reinforced auxetic honeycomb for short). We investigated and compared this novel unit cell design against a wide parameter range. Compression tests were carried out on specimens 3D-printed with a special, unique, flexible but tough resin mixture. The results showed that the additional, centrally pronounced reinforcements resulted in increased deformation stability; parameter-independent, non-buckling deformation behaviour is achieved; however, the novel structure is no longer auxetic. Mechanical properties, such as compression resistance and energy absorption capability, also increased significantly—An almost four times increase can be observed. In contrast to the deformation behaviour (which became predictable and constant), the mechanical properties can be precisely adjusted for the desired application. This novel structure was also investigated in a highly accurate, validated finite element environment, which showed that critical stress values are formed in well-supported regions, meaning that critical failure is unlikely. Our novel lattice unit cell design elevated the auxetic honeycomb to the realm of modern, high performance and widely applicable lattice structures.

## 1. Introduction

Cellular materials are the future of lightweight and efficient engineering part design, as these materials can achieve special properties through their microstructural design [1,2]. Owing to their unparalleled mechanical [3,4,5,6], thermal [7], optical [8] and electromagnetic [9,10] properties, these materials can and should be used in a wide range of applications. Cellular materials can either be stochastic or periodic [11]; natural materials are mostly stochastic (with a handful of exceptions [12,13]), such as wood and bones [14], while periodic cellular materials are mostly man-made, such as metamaterials. 

Metamaterials can be classified in several ways based on their properties of interest. If the electrodynamics of a substance are considered, metamaterials can be grouped based on signs of permittivity and permeability [15]; this classification was introduced by Veselago [16]. In terms of mechanical properties, metamaterials are also classified based on the sign of their Poisson’s ratio. Based on their deformational behaviour under large strain loads, metamaterials can either have compressive shrinkage or compressive expansion, or no lateral deformation is present. Based on the foregoing, metamaterials can be classified into three groups: negative Poisson’s ratio (NPR), positive Poisson’s ratio (PPR) and zero Poisson’s ratio (ZPR) [17]. NPR, PPR and ZPR metamaterials all have their advantages and specific applications. Positive Poisson’s ratio materials are most widely used for lightweighting [18], owing to their high strength-to-weight ratio, and where tuneable mechanical properties [19] are required. The most iconic PRR lattice is the honeycomb structure, also referred to as a hexagon lattice [20].

Zero Poisson’s ratio materials are a less researched area, but there are some applications where only they can be used. ZPR materials have a promising future in the aerospace industry for aircraft wing morphing [21]. The Poisson’s ration of many native tissues is also zero [22], thus ZPR metamaterials are promising in tissue engineering [23].

In recent years, technological advances in additive manufacturing (AM) and market demand [24,25] have fuelled the research of periodic cellular NPR materials, due to their outstanding mechanical properties [26,27,28,29,30,31] and unique deformation behaviour. Materials with a negative Poisson’s ratio shrink laterally when compressed, contrary to conventional materials [32,33]. These structures are referred to as auxetics (named by Evans et al. [34]). The history of auxetic structures dates back to 1927 [35]. The growth of additive manufacturing for fabricating auxetics has been incremental [36]. Auxetic structures benefit from high manufacturing accuracy; thus, laser-based additive technologies are used to manufacture these parts. The recent robotisation of such technologies has greatly improved the productivity, quality (through efficient control) and dimensional accuracy of metallic auxetics [37]. Curved AM is another recent breakthrough, which enables the quick production of curved shells with minimal waste [38]. Auxetic shells are of interest in biomedical applications, such as pedicle screws [39,40].

One of the most widely used and researched structures is the re-entrant hexagonal honeycomb, which has a re-entrant deformation mechanism [30]. The in-plane behaviour (characterised by Poisson’s ration, Young’s modulus etc.) of the structure has been described by several researchers [41,42,43]; thus, the effect of several geometrical parameters [44] is widely understood. All auxetic deformation mechanisms require substantial porosity to reap their benefits, thus their in-plane stiffness is limited. Consequently, such a structure is not recommended for great load-bearing requirements [45]. 

One of the most significant drawbacks of re-entrant structures is their tendency to buckle [30]. It increases as the relative density of the structure decreases [14], which poses a limit to lightweighting. Xiuhui et al. [46] theoretically described (based on classical beam–column end-moment behaviour expressed in a matrix form) the buckling mechanism of the re-entrant honeycomb structure and were able to determine its global and local buckling mode. They found that global buckling is more likely than local buckling. Mirsalman et al. [47] found that certain geometrical modifications, such as changing the aspect ratio, have little to no effect, while increasing the thickness-to-length ratio influences the critical buckling load. It can be concluded that only changing certain geometrical parameters of the re-entrant honeycomb structure will not increase its in-plane stiffness and buckling strength enough to make it a high-performance structure. 

The core concept of metamaterial engineering is to artificially create structures with the desired properties, but researchers have also developed novel or improved designs based on existing lattice structures. In this paper, we improve the deformation behaviour of the doubly re-entrant auxetic honeycomb structure while retaining its outstanding mechanical properties. Researchers use two approaches to improve the properties of the auxetic honeycomb structure—they can either replace components or add components [48], which is also referred to as the nested geometry approach. In this article, we also aim to enhance the mechanical and deformational properties by creating a novel unit cell design based on the nested geometry approach. 

**Figure 1:**
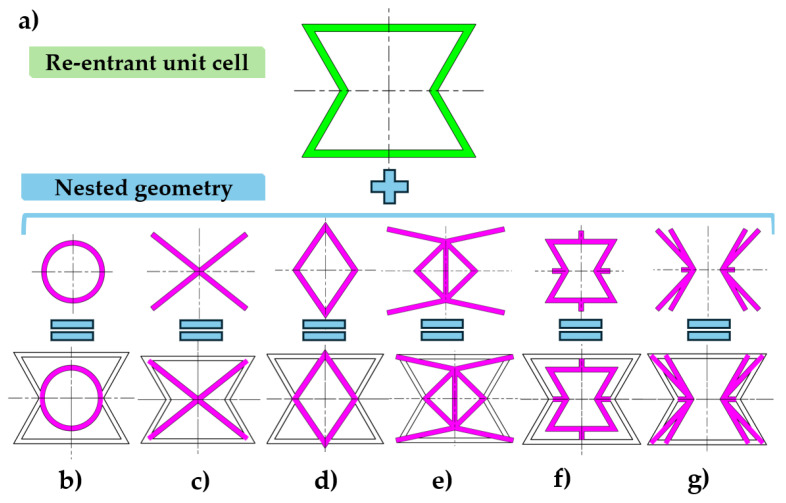
Nested geometry approach to improving the re-entrant unit cell design. (**a**) The original re-entrant unit cell. (**b**) Elliptical annular structure based on [48]. (**c**) Diagonally connected corner design based on [49]. (**d**) Added rhombus based on [50]. (**e**) Complex reinforcement design using simple trusses based on [51]. (**f**) Offset self-similar inclusion based on [52] (**g**) Re-entrant edge offset self-similar design [53].

Difeng et al. [48] introduced an annular elliptical structure in the centre of the unit cell (Figure 1b). This design provides further connections between the horizontal and the diagonal edges of the unit cell, providing increased support without affecting lateral deformation; as a result, this modification only slightly affects the structure’s Poisson’s ratio. 

Fu et al. diagonally connected the corners of the unit cell (Figure 1c), which resulted in increased Young’s modulus [49]. The diagonally reinforced structure retained its auxetic behaviour, but at large deformations, it became a PPR material. Researchers have also used a different but still diagonal (rhombus) arrangement (Figure 1d) [50]. This layout improved in-plane stiffness and reduced the porosity of the structure. However, reduced porosity might hinder auxetic behaviour; thus, in their article they emphasised that the newly added rhomboid edges should be thinner. Instead of the edges of the auxetic honeycomb, the thinner rhomboid edges will buckle during compression, resulting in the planned improvement in in-plane stiffness and critical buckling strength and retained auxetic behaviour. Nedoushan et al. [51] added several extra edges to the unit cell, resulting in a highly complex structure (Figure 1e). These added segments predictably resulted in increased strength and a tuneable Poisson’s ratio. Deformation stability can also be significantly increased with a special type of nested geometry: self-similar inclusion [52,53,54] (see Figure 1f,g).

The novel structures listed above show that each modification affected the Poisson’s ratio, even resulting in positive Poisson’s ratio values in some cases. Moreover, the advantageous mechanical properties of the auxetic honeycomb structure remained, so by sacrificing the auxetic behaviour but retaining the re-entrant deformation behaviour with the proper geometrical reinforcement, high-performance lattice structure designs can be produced. Grima et al. created [17] a lattice structure with a zero Poisson’s ratio, which is based on the re-entrant honeycomb; they created a semi-re-entrant honeycomb. This is a perfect example of utilising a certain behaviour mechanism (i.e., the re-entrant mechanism) to create the desired properties.

Gradient design can be considered as the third group of metamaterial engineering. Gradient design is mainly aimed at improving mechanical properties [55,56] by varying the nominal lattice thickness throughout the specimen. Tomita et al. successfully implemented gradient design for controlling the buckling behaviour of origami-based auxetic structures [57].

Finally, it is important to study lattices cost-effectively, fast and with a high accuracy; thus, a well-established finite element environment is key, similarly to technical creations [58], construction problems [59,60] or thermoelectric problems [61], for example. 

The literature review above presented several stabilising modifications for the re-entrant honeycomb structure, but in this article, our goal is to eliminate buckling and further improve the properties of the doubly re-entrant lattice structure proposed in our previous study [62], ultimately resulting in a structure where only mechanical properties depend on geometrical parameters. The doubly re-entrant lattice structure is also based on the original re-entrant lattice structure; hence the researched methods can be useful, especially the diagonal reinforcements. 

## 2. Materials and Methods

In this section, the derivation of the novel unit cell is introduced, alongside the dimensions of the main unit cell and the specimen. Two parameters, whose effect is important, are also introduced in this section. The introduction of the novel unit cell is followed by describing the fabrication process of real-life test specimens. Real-life compression testing is used for validation, mechanical properties are determined with finite element method (FEM) simulations, and the complex FEM test environment is also introduced in this section. Then, compression testing is used for validation of the results. A unique resin mixture is used for the production of the specimens, whose FEM material model setup is also presented in this section. This section ends with the introduction of the test environment. 

### 2.1. Unit Cell Design

In this paper, a novel modified unit cell design is proposed with a nested geometry. Our aim was to eliminate the parameter-dependent deformation behaviour of the doubly re-entrant unit cell design [62], so that no buckling occurs, regardless of the design of the unit cell, and only the mechanical properties depend on the parameters. This way, one can implement this lattice into a design without any substantial simulation.

In our previous study, where we proposed the doubly re-entrant unit cell design, some parameter combinations resulted in buckling. The stabilisation design guidelines presented in our previous paper [62] indicated that thin vertical segments are to be avoided, and one must create prominent centre segments to avoid buckling. As far as buckling is concerned, the centre segment of the unit cell is crucial; hence in this paper, we aim to eliminate parameter-dependent buckling behaviour by further improving the geometrical design of the centre segment. 

**Figure 2:**
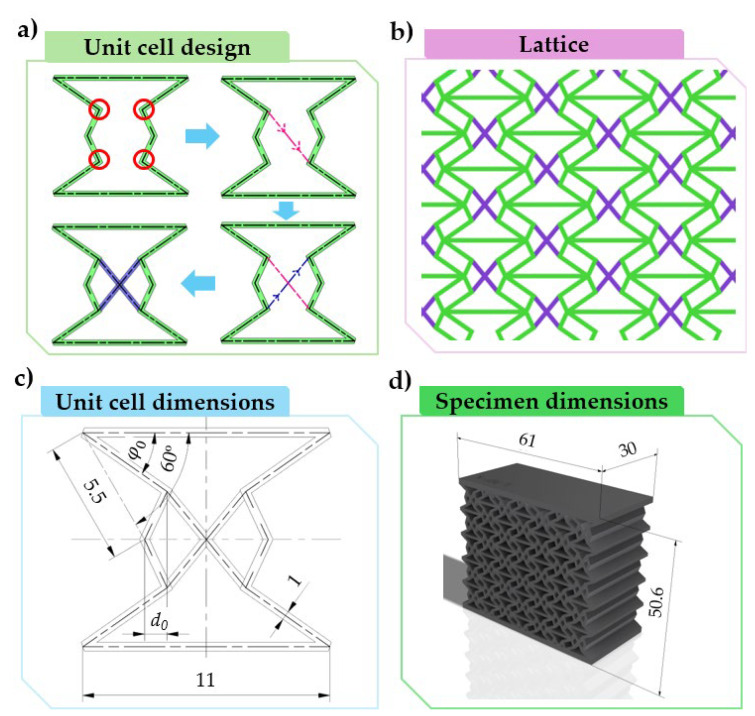
The novel unit cell. (**a**) Derivation of the novel x-reinforced unit cell design based on the doubly re-entrant unit cell design. (**b**) A lattice pattern made of the x-reinforced unit cell design. (**c**) The dimensions of the main unit cell and the specimen in millimetres. (**d**) A 3D CAD representation of a specimen to be tested, made up from the novel unit cell design, including the enclosing dimensions.

A more prominent centre section can be produced by diagonally connecting the four new breakpoints of the doubly re-entrant unit cell, resulting in an “*x*” shape (referred to as *x-reinforcement*) (Figure 2a). This novel nested design stabilises the centre of the unit cell, as the two newly added edges intersect at exactly the centre of the unit cell. The addition of the two new edges, i.e., the “*x*” shape, predefines a symmetrical, non-buckling deformation, as all edges of the unit cell are interconnected and constrained to one another. Furthermore, this added “*x*” shape follows the path of the upper re-entrant edges for an even more stable design, where the course of deformation is predefined. With our embedded design, one of our aims was to create a simple geometrical modification which reinforces the unit cell, yet does not limit its lattice-like behaviour, i.e., the interaction between unit cells; hence, no continuous horizontal or vertical lines would have been acceptable. Figure 2b shows a lattice made up from these x-reinforced unit cells, on which there are no continuous horizontal or vertical edges that would limit the lattice-like behaviour.

In this paper, we investigated the effect of the imbedded “*x*” shape on the same parameter range used earlier for a conclusive study [62]. Figure 2c shows the dimensions of the main unit cell, and the parameters *offset* ($d_{0}$) and *deg* ($\varphi_{0}$). These parameters, alongside specimen designations, are listed in Table 1. The effect of “*x*” reinforcing is compared to the original specimen design; hence, both reinforced and original specimens form the scope of our study. Reinforced specimens are distinguished by an “*x*” added to the specimen designation (i.e., 0630X), while original specimens are denoted as “*ORIG*” (i.e., 0630ORIG). 

We investigated the efficiency of our design by measuring and comparing its mechanical properties against the original design. Mechanical properties such as compressive force and absorbed energy are obtained by compression testing. For an accurate and expandable representation, it is not the unit cells, but specimens (see Figure 2d) made up of neighbouring unit cells forming a lattice, that are subjected to compressive load. Based on our previous research [62,63], a specimen consisting of 5 by 7-unit cells can accurately characterise the properties of the unit cells.

### 2.2. Specimen Fabrication

Specimens were fabricated with a highly accurate additive manufacturing (AM) technology, vat polymerisation–masked stereolithography (MSLA). Specimens were printed on an Anycubic Photon M3 Printer (Hongkong Anycubic Technology Co., Limited, HongKong, China). The printing parameters are listed in Table 2. 

A unique, flexible yet tough resin mixture was specifically created for these specimens. Based on our experiments, a mixture of 80% Litliq FX 60 (black) TPU-like resin and 20% Litliq TH 50 (blue) tough resin allowed flexible deformation behaviour without significant cracking. Our aim was to investigate deformation behaviour over a large deformation range without specimen failure, hence the unique resin mixture. 

Specimens were removed from the build plate via a stainless steel scraper (with a blade). The removed objects were cleaned in a recirculated isopropyl alcohol (99.8% purity) tank for 5 min, followed by a 15-min resin-hardening cycle in an Anycubic Wash and Cure 2.0 25 W device (Hongkong Anycubic Technology Co., Limited, HongKong, China).

### 2.3. Finite Element Modelling

**Figure 3:**
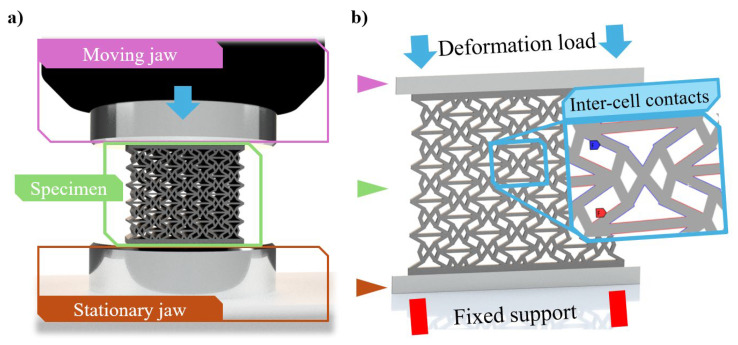
The finite element model. (**a**) CAD representation of a real compression testing environment. (**b**) Finite element method representation of the compression testing environment.

Compressive finite element method (FEM) simulations were carried out to acquire the mechanical properties of each parameter combination listed in Table 1. FEM simulations were validated with the compression testing presented in Section 2.5. For accurate results, the necessary elements (i.e., the moving and stationary jaws of an imaginary compression testing machine) of the compression testing environment also formed part of the FEM model. Figure 3a shows the CAD representation of a compression testing environment, which will be simplified to a FEM model as follows (Figure 3b). 

Based on our previous results [62,63,64] and the geometrical ratios of the specimen, two-dimensional plane strain simulations are accurate enough. The moving and stationary jaws were simplified as rigid rectangular bodies, but the specimens were not simplified. Large-deformation FEM simulations will be carried out on the specimens, hence contact between certain edges is inevitable, so accurate contact definition is required. Frictional contact was defined between the specimen and the jaws of the compression testing machine characterised by a friction coefficient of 0.3 [65]. Frictional contact was set between every possible contacting edge inside the specimen with a friction coefficient of 0.17 [66]. The contact detection method was set to Nodal-normal to target, and a slight (0.02 mm) penetration tolerance was allowed inside the specimen to aid with convergence. FEM simulations were carried out until the onset of compaction, i.e., as long as lattice-like behaviour was present. Edges started to overlap at 20 mm deformation on average, indicating the end of lattice-like behaviour. 

As for the boundary conditions, fixed support was applied to the bottom edge of the stationary jaw. The load was deformation-controlled, applied in one step to the upper edge of the mowing jaw. Large deformations were turned on, and substeps were set to achieve convergence. 

**Figure 4:**
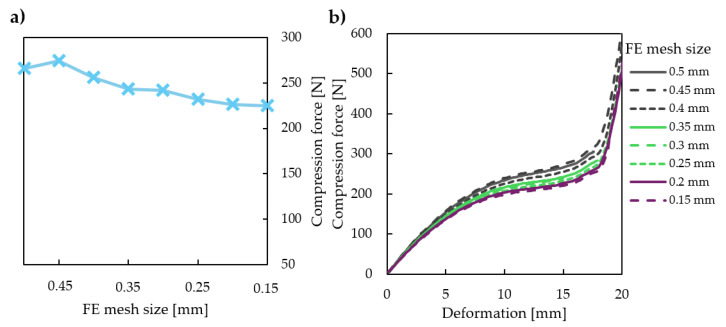
Mesh convergence study carried out on a specimen characterised by $d_{0}=1\;\mathrm{mm}$ and $\varphi_{0}=40{}^{\circ}$. (**a**) Compressive force at 10 mm deformation with different mesh sizes. (**b**) Force–displacement curves recorded with different mesh sizes in the 0–20 mm deformation range.

A mesh convergence study was conducted on a specimen characterised by $d_{0}=1\;\mathrm{mm}$ and $\varphi_{0}=40{}^{\circ}$ to obtain the optimal mesh size. The mesh convergence study results are plotted in Figure 4; Figure 4a shows the maximum compressive force at 10 mm deformation, while Figure 4b shows the force displacement curve on the studied 0–20 mm range at different mesh sizes. Increasing the mesh size is essential, as a coarse mesh results in unreliable results and insufficient convergence (Figure 4b). Our results indicated that refining the mesh beyond 0.2 millimetres is not justified. Specimens were meshed with fine, 0.2 mm linear elements with the quadrilateral dominant method. 

### 2.4. Finite Element Material Model

To investigate the deformation behaviour of the geometry over a wide range of measurable forces, we created a unique, flexible yet tough material mixture (see Section 2.2). The properties of this unique material mixture are required for FEM simulations. The main constituent of the mixture is a flexible TPU-like resin; therefore, a hyperplastic material model should be used. It is crucial to map the type of material model required in advance, as the model requires real-life material tests. Hyperplastic material models in general rely on a series of experimental data inputs, and the FEM software calculates the parameters of the hyperplastic material model by curve fitting to experimental data, such as uniaxial, biaxial and shear test data.

Curve fitting can be performed to a single uniaxial test, but in this case, the obtained hyperplastic material model would be highly inaccurate [67]. Based on our experience and the cited literature, at least two types of experimental data have to be used for an accurate hyperplastic material model. Our hyperelastic material model will be based on uniaxial tension test data (tensile testing) and equivalent biaxial tension test data (compression testing).

**Figure 5:**
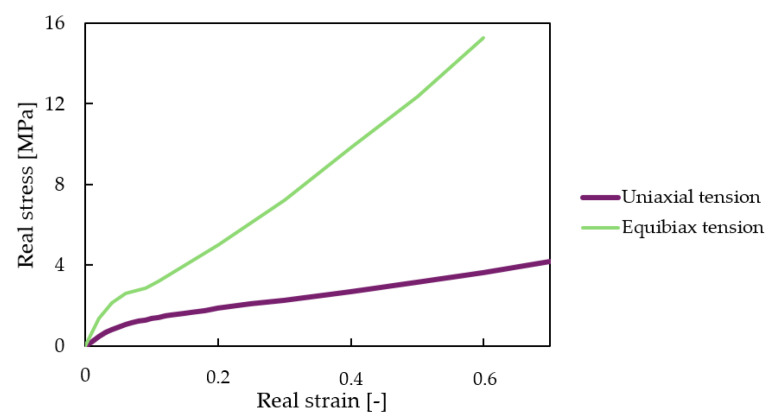
Real stress–strain values for the finite element material model; uniaxial tension and equivalent biaxial tension (compression) real stress–strain data sets are plotted.

A series of tensile tests were conducted on printed specimens according to EN ISO 527-2 [68], with a Zwick Z020 (ZwickRoell GmbH and Co. KG, Ulm, Germany, Europe) tensile testing machine with a measuring limit of 20 kN. The tensile specimens were prepared with a thickness of 4 mm (as per the standard), and thicker, 6 mm versions were made according to the standard as well. The evaluated real stress–strain curves are in Figure 5. Equivalent biaxial test data can be obtained with high accuracy by compression testing, for incompressible materials [69]; biaxial tensile test data and uniaxial compression test data have the same magnitude, but a sign conversion is needed. Even though this is only an approximation (albeit a highly accurate one) of the real biaxial test data, the inclusion of equivalent biaxial tension test data makes any hyperelastic material model more accurate. Figure 5 shows converted real stress–strain equivalent biaxial tension data. Simulations were carried out in Ansys Workbench 2024 R1. Uniaxial and equibiaxial measurement data were imported. A new material model was created, and a two-parameter Mooney–Rivlin (derived by Mooney [70] and expressed by Rivlin [71]) model was fitted to the measurement data set with good agreement. The remaining material properties were provided by the resin manufacturer (Dongguan Godsaid Technology Co., Tangxia Town, Dongguan, Guangdong, China). Table 3 shows the calculated and known material properties. 

Structural steel material was assigned to the moving and stationary jaws of the compression testing machine. Table 3 also shows the properties of the structural steel.

### 2.5. FEM Result Validation 

**Figure 6:**
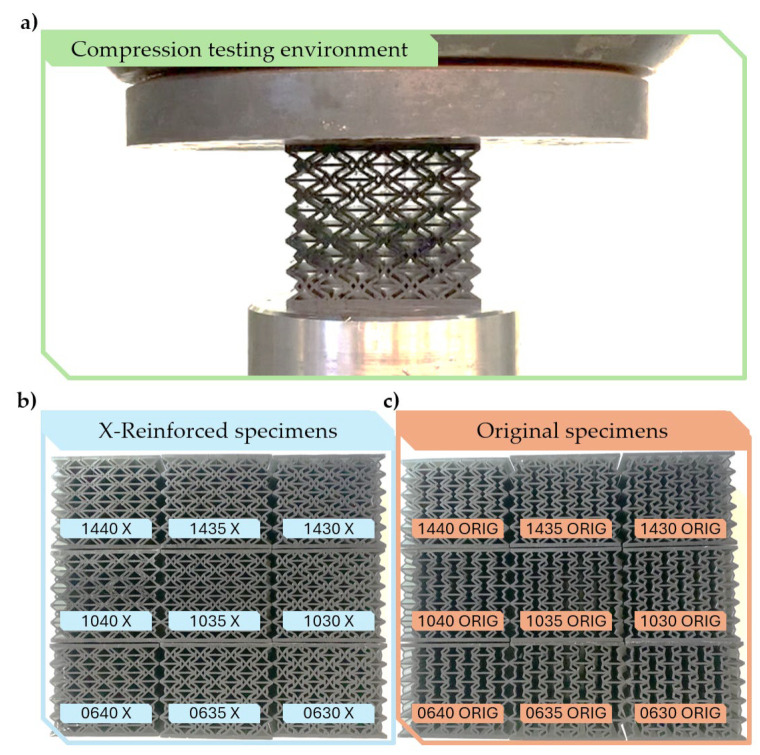
Actual compression testing. (**a**) The compression testing environment; (**b**) all 9 X-reinforced specimens; (**c**) all 9 original specimens.

The finite element method simulations were validated by real-life compression testing. All 9 x-reinforced and original specimens characterised by parameters listed in Table 1 were compressed. Measurements were conducted on a Hegewald and Peschke 40-ton capacity machine at a low speed of 5 mm/min up to 20 mm deformation. Figure 6a illustrates the compression testing environments, and Figure 6b,c show all 9 additively manufactured x-reinforced and original tested specimens. 

## 3. Results

In this section, the finite element compression test results are presented and evaluated in the forms of force–displacement curves and deformation images at different deformation levels. Absorbed and specific absorbed energy values are also calculated and plotted; the results are explained in detail, and the underlying mechanical and deformational phenomena are also discussed. The effect of the parameters is discussed and plotted in three dimensions. The FEM results are also validated in this section. The validated FEM results form the basis of further evaluations, such as calculating the Poisson’s ratio. The section ends with comparing the stress distribution of the original and x-reinforced specimens. 

### 3.1. Compression Test Results

**Figure 7:**
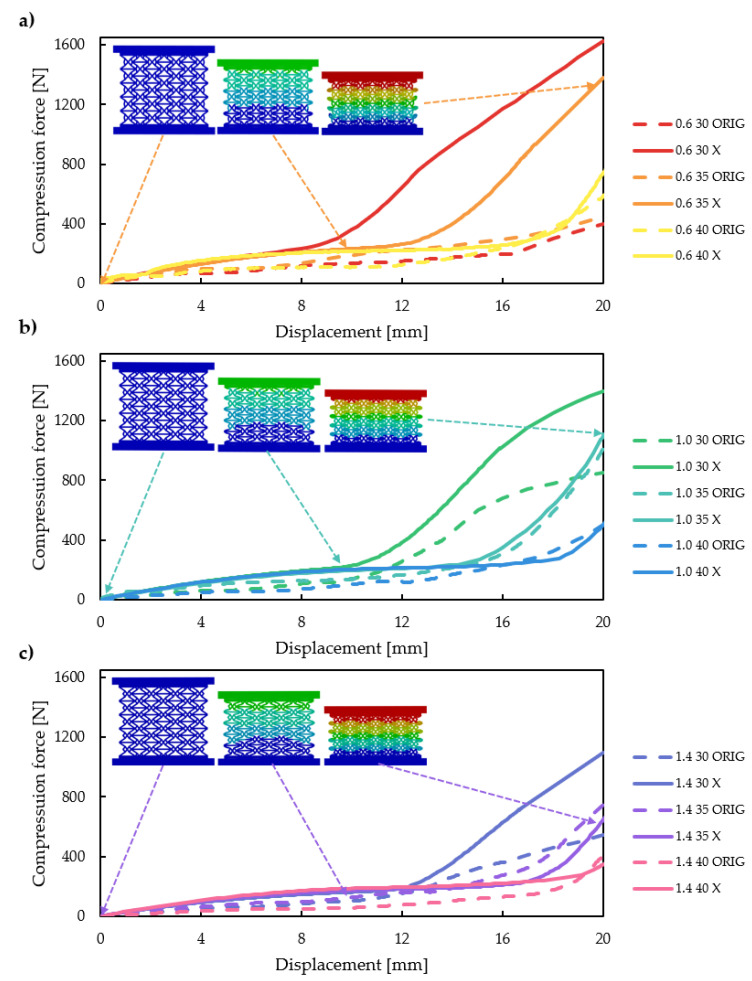
Force–displacement curves acquired from finite element simulations. (**a**) Force–displacement curves for *offset* = 0.6 mm. (**b**) Force–displacement curves for *offset* = 1.0 mm. (**c**) Force–displacement curves for *offset* = 1.4 mm.

**Figure 8:**
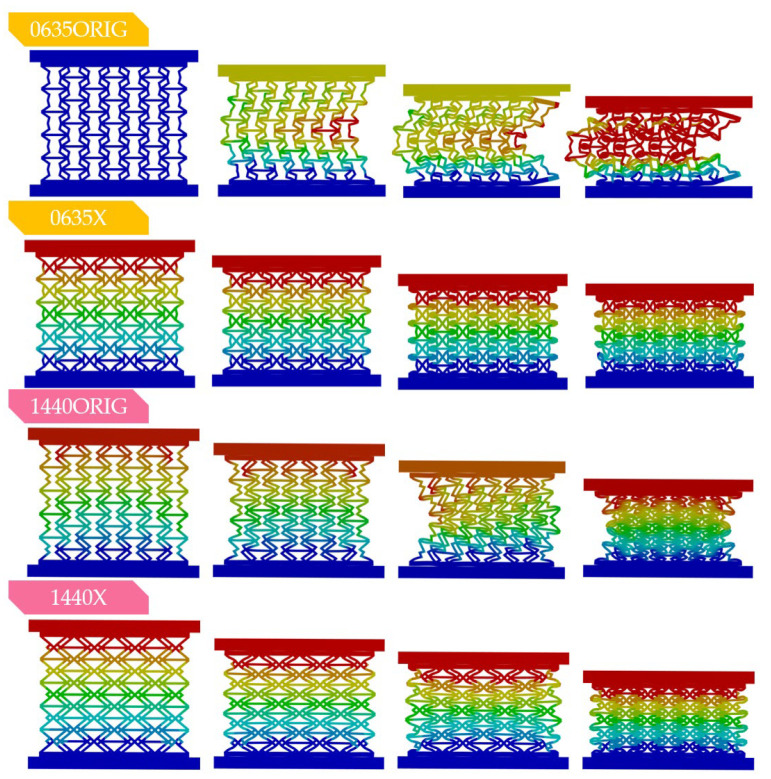
Comparing the deformation behaviour of the original and x-reinforced specimens. One original specimen (1440ORIG) is prone to buckling, while the other original specimens show continuous auxetic behaviour.

The finite element method compression test results on the 0–20 mm range are plotted in Figure 7a-c separately for all *offset* values. Maximum compressive force values are also plotted on three-dimensional curves in Figure 10 as a function of certain parameters. The deformation behaviour is of the greatest interest, being the main motive behind this novel design; deformed shapes at different strain levels are plotted in Figure 8. 

At first sight, both the force–displacement and deformation results are promising. The force–displacement curves (Figure 7) of the reinforced specimens surpass the curves of the original specimens in almost all cases, and also, the spike in the force–displacement curves occurs at lower deformation values. X-reinforced force–displacement characteristics also appear more stable and indicative of higher absorbed energy in almost all cases. Deformation seems to be even throughout the entire load and specimen as well, with no sudden compaction either on the force–displacement curves or on the deformed images (Figure 8). Figure 8 compares the deformation behaviour of two original and reinforced specimens. Buckling occurs in the original specimens, while none of the x-reinforced specimens buckle. The most notable difference in the force–displacement curves between the original and x-reinforced specimens is observed for specimens 0630, 0635, 0640 and 1030 (Figure 7a,b). This is explained by the improvement in deformational behaviour; these four specimens buckled laterally with the original design, but owing to the x-reinforcement, buckling does not occur with any parameter combination. The deformation behaviour is explained in detail in Section 3.4.

**Figure 9:**
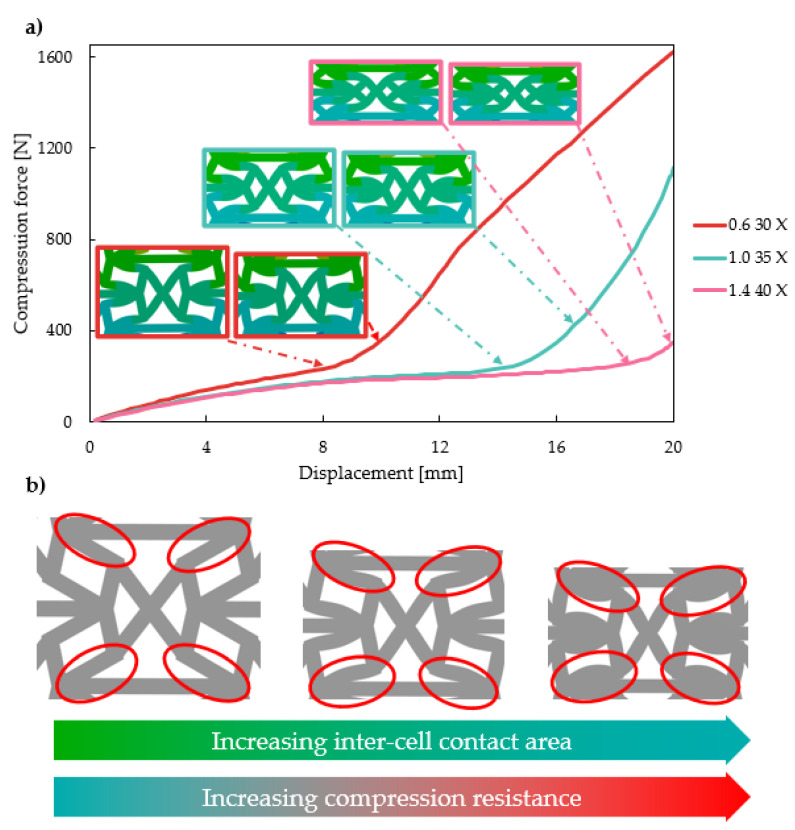
Two segmented force–displacement characteristics. (**a**) Force displacement curves for different initial parameter values. (**b**) Inter-cell deformation explaining the two-segmented force–displacement phenomenon.

The force–displacement curves of the reinforced specimens show a stable steady increase with no sudden significant regression, which would be indicative of unpredictable deformation behaviour, such as buckling. The steady increase in low forces at the initial stage is followed by a still steadily increasing segment, reaching high forces. This two-segment force–displacement characteristic is explained by the changes in inter-cell deformation. Figure 9a presents three curves, which show a change in characteristics at different deformations. Unit cell deformations are also presented in Figure 9a at these notable points and a 2 mm deformation later. Based on Figure 9a, one can clearly see that in all cases the sudden change in characteristics is due to the same inter-cell deformation stage. As the four upper re-entrant edges (Figure 9b) start to contact the horizontal edges, the inter-cell contact area grows rapidly. Figure 9a shows that only a small (2 mm) deformation after the initial contact results in a large contact area between these edges. This contact area increases with deformation, which results in increasing compression resistance. The contact pair is a stable contact pair, unlike in the original re-entrant honeycomb or the doubly re-entrant honeycomb unit cell [62]. Once in contact, separation cannot occur, as the x-reinforcement limits outward and inward motion.

**Figure 10:**
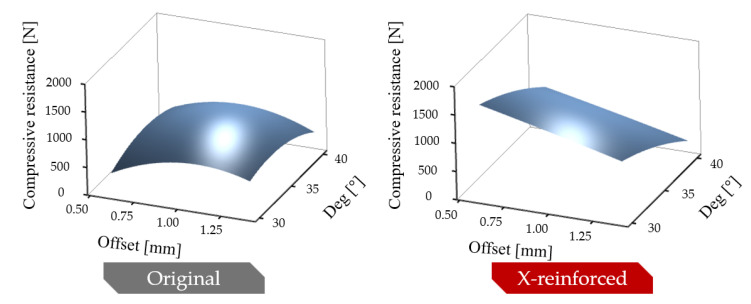
Effect of initial geometric parameters on maximum compressive forces.

The initial geometrical parameters (*deg* and *offset*) greatly affect the onset of force increase. Figure 7a–c and Figure 9a indicate that increasing the *deg* parameter value pushes the force increase to greater deformations, resulting in lower maximal compression forces. The phenomenon meets our expectations, as initial contact between these two edges solely depends on the enclosed angle, which is the *deg* parameter (see Figure 2c). The effect of the *offset* parameter is not obvious based on Figure 7; thus, the effect of the parameters on the maximal compression force is plotted in 3D (Figure 10). Figure 10 confirms our previous observations; that is, increasing the value of the *deg* parameter increases the maximal force. On the other hand, with x-reinforced specimens, decreasing the *deg* and *offset* parameters increases compressive resistance in the investigated parameter range.

Based on the characteristics of the curve alone, it can be stated that x-reinforcement is indeed beneficial, resulting in high-performance behaviour. 

### 3.2. Absorbed Energy

**Figure 11:**
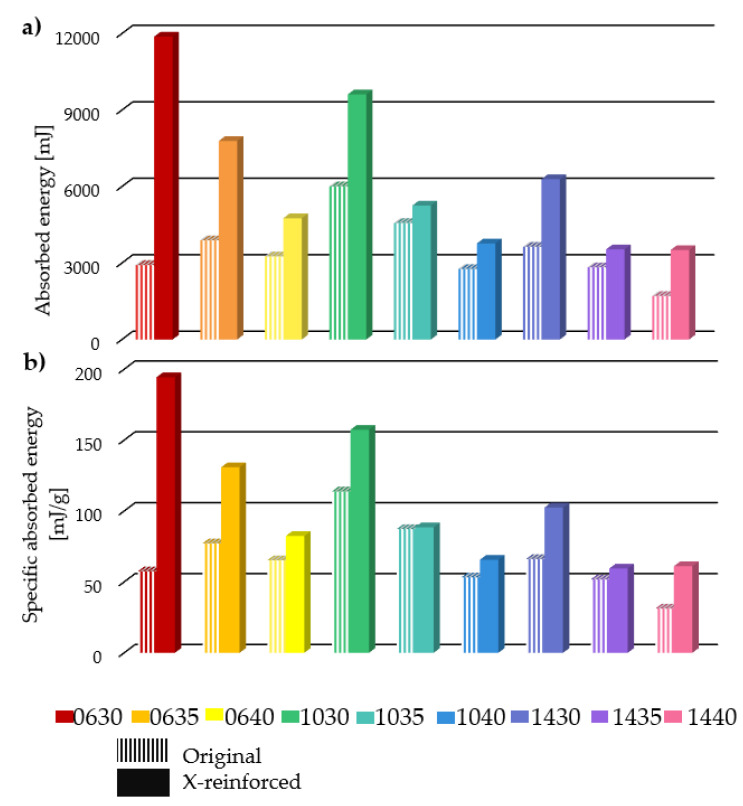
Absorbed energy. (**a**) Absorbed energy of each original and x-reinforced specimen. (**b**) Specific absorbed energy of each original and x-reinforced specimen.

Absorbed energy and specific energy are plotted in Figure 11a and b, respectively. The added reinforcement segment adds to the weight of the specimen, so the results must also be considered as weight-specific values. Figure 11a,b clearly indicate that reinforcement improves the energy absorption capability of each parameter variation, even when specific absorbed energy is considered. Absorbed energy is calculated from force–displacement curves; absorbed energy is defined as the area under the force–displacement curve. A close relationship between the characteristics of the force–displacement curve and absorbed energy is expected. Lower *deg* parameter values with constant *offset* values result in greater forces, and the force growth phase starts at lower deformations (Figure 7 and Figure 9). It induces higher energy, as expected. Lower *deg* values suggest greater energy absorption capabilities. 

**Figure 12:**
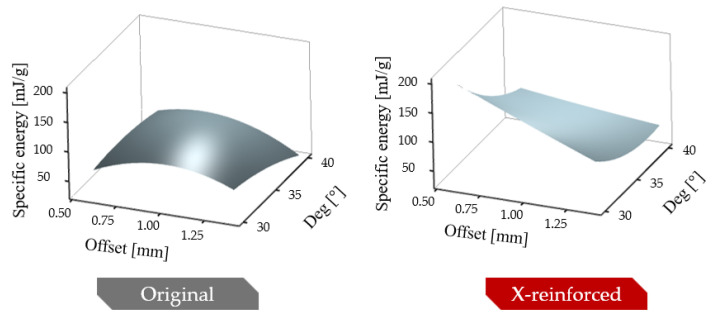
Effect of initial geometric parameters on specific absorbed energy.

Similarly to the maximal compressive forces, x-reinforcement is beneficial. In the studied parameter range combination, all reinforced specimens surpassed the energy absorption capability of the original specimens. The parameter dependency of the absorbed and specific absorbed energy is plotted on 3D graphs in Figure 12. 

### 3.3. Validation of FEM Results 

**Figure 13:**
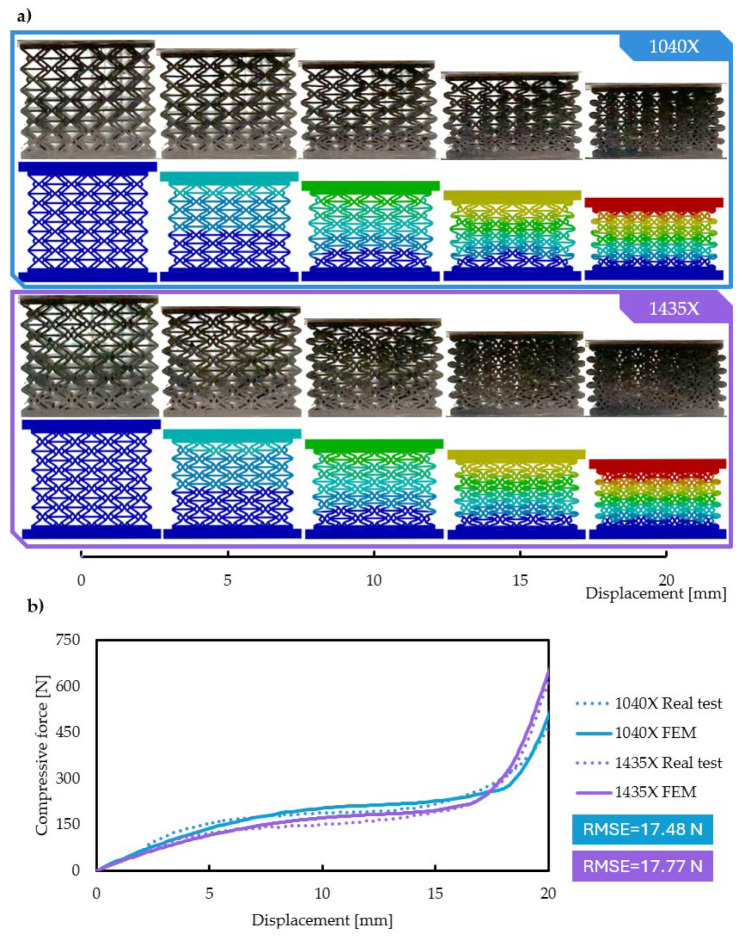
Validation of finite element method results. (**a**) Comparing deformed shapes at different strains. (**b**) Compressive force in FEM and actual compression tests.

The accuracy of the established finite element method test environment was validated via comparing the obtained results to actual compression test results. All nine (original and x-reinforced) specimens were additively manufactured and subjected to compression testing. The FEM results are in good agreement with the real test data. Figure 13b compares the force–displacement curves, while Figure 13a compares deformed shapes with different displacements. 

The deformed shapes in the FEM and actual compression tests are in good agreement on a local (i.e., unit cell) and global (specimen) level. The observed force displacement curves are in good agreement. The root mean square error (RMSE) was calculated between the FEM and experimental results. The RMSE values are low (2.8% of the maximum force value), quantifying an accurate level of agreement; the values are plotted in Figure 13. The established FEM test environment can be deemed accurate enough, which is mostly due to proper contact and material model definition.

### 3.4. Deformation Behaviour and Poisson’s Ratio 

The main aim of our recent x-reinforced design was to produce a parameter-independent behaviour mechanism. In our previous research, we concluded that both the re-entrant and the novel doubly re-entrant honeycomb buckle laterally, even though the doubly re-entrant design was greatly improved [62]. For a truly widely applicable lattice design, it must be user-friendly. A lattice in our interpretation and that of the industry is considered user-friendly if the mechanical properties change when certain prescribed parameters are changed with known outputs (detailed within the design guidelines) and deformation behaviour is not parameter-dependent (i.e., buckling will not occur). The deformation behaviour for all parameter combinations is listed in Table 4. Table 4 and the deformation images in Figure 8 and Figure 13 clearly indicate our nested x-reinforced design fulfils our basic goal. The reinforced specimens have parameter-independent deformation behaviour. Table 4. also compares the deformation behaviour of the doubly re-entrant and the novel x-reinforced design. 

**Figure 14:**
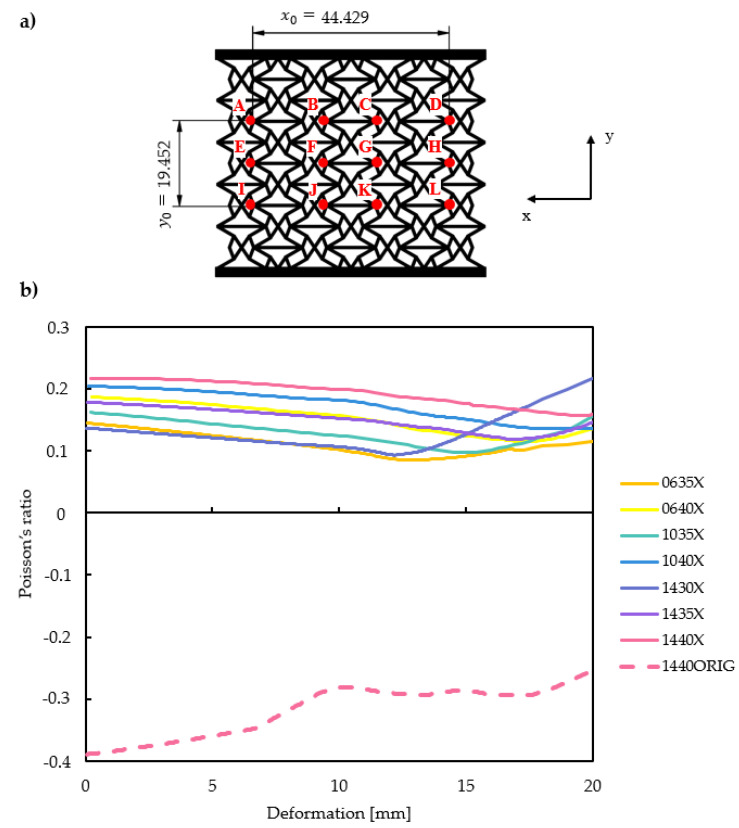
Calculating the Poisson’s ration of X-reinforced specimens. (**a**) Points used to retrieve deformation components to calculate their Poisson’s ratio; (**b**) calculated Poisson’s ratio for several X-reinforced specimens compared to the original doubly re-entrant specimen.

Contrary to the original, doubly re-entrant structure, the x-reinforced structure does not seem to be auxetic. Poisson’s ratio was determined from twelve measurement points in the middle of the specimen, based on Yang et al. [72], as shown in Figure 14a. Deformation components in both the *X* and *Y* direction were obtained from the FEM model. Poisson’s ratio is the negative quotient of the transverse and axial strain; see Equation (1).

(1) $$\mu =-\frac{\varepsilon_{x}}{\varepsilon_{y}}$$

During compression, the transverse strains are denoted as $\varepsilon_{AD}$; $\varepsilon_{EH}$; $\varepsilon_{IL}$; the axial strains are denoted as $\varepsilon_{AI}$; $\varepsilon_{BJ}$; $\varepsilon_{CK}$; $\varepsilon_{DL}$. The average of the transverse strain was taken as the x-directional strain $\varepsilon_{xAVG}$, while the average of the axial strain was taken as the y-directional strain $\varepsilon_{yAVG}$ (Equations (2) and (3)).

(2) $$\varepsilon_{xAVG}=\frac{\varepsilon_{AD}+\varepsilon_{EH}+\varepsilon_{IL}}{3}$$

(3) $$\varepsilon_{yAVG}=\frac{\varepsilon_{AI}+\varepsilon_{BJ}+\varepsilon_{CK}+\varepsilon_{DL}}{4}$$

It is of interest to plot Poisson’s ratio as a function of compressive load, i.e., displacement. Poisson’s ratio is plotted for several specimens in Figure 14b. 

Contrary to the original doubly re-entrant structure (bottom of Figure 14b), the reinforced structures have a low but positive Poisson’s ratio. The Poisson’s ratio is 0.144 on average; the lowest obtained is 0.08 for the 0635X specimen. Compaction starts at 20 mm on average (for specimens with *deg* values of 30 degrees, compaction starts at a lower deformation), which appears as an increase in Poisson’s ratio. The results are in line with expectations; although the nested “x” reinforcement has a positive, stiffening and stabilising effect, it inhibits inter-cell auxetic deformation, hence the positive Poisson’s ratio. 

Although their Poisson’s ratio is positive, one of the greatest advantages of auxetic structures, the ability to absorb a significant amount of energy, remained and even surpassed that of the original auxetic honeycomb lattice (Figure 8). On the other hand, an increased Poisson’s ratio results in a reduced shear and fracture resistance [73], which must be taken into consideration for applications with a high number of cyclic loads.

Finally, a comparison of the characteristics of a reinforced and a non-reinforced structure (Figure 14b) shows that the reinforced structure has a more constant Poisson’s ratio over the deformation spectrum, making it superior for a wide range of applications. 

### 3.5. Stress Distribution

**Figure 15:**
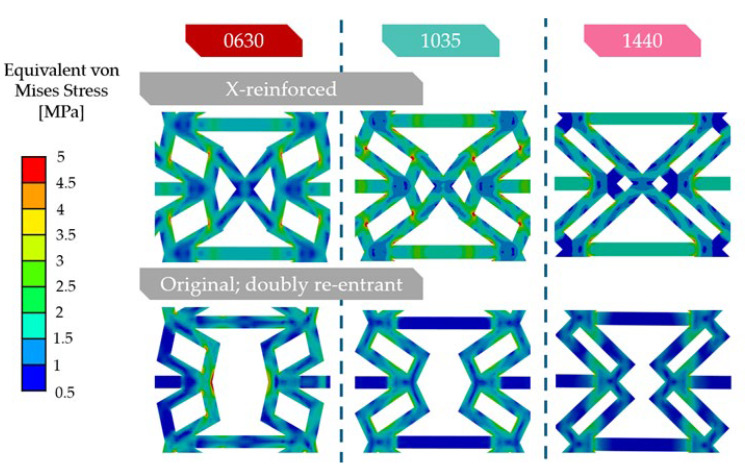
Comparing the stress distribution of original and x-reinforced specimens.

Finite element method stress results can indicate potential failure points; Figure 15 compares the equivalent von Mises stress values for the original and the x-reinforced structure at 20 mm deformation. In Figure 15, stress is plotted on the undeformed unit cell for clarity. As presented in Figure 15, the doubly re-entrant structure has an even stress distribution; no significant local stress regions appear that would indicate failure or crack formation. On the other hand, in the x-reinforced structure, localised high-stress regions can be observed at some corners. These corners are the corners that are connected by the “x” reinforcement, which has a limiting effect on the original auxetic deformation, hence the difference in stress distribution. These localised stress regions can be a source of failure points, so it is a drawback compared to the original structure. Even though cracks may appear in these regions (depending on the base material), the resulting weakening would not be critical, since these edges on which fractures may occur are supported at both ends through the x-reinforcement. 

Real compression tests did not cause specimen failure for the reinforced group; regardless, these corner points should be regarded as potentional failure points in the case of large deformations. Even though the stress distribution in the original structure is more even, for specimens with buckling behaviour (Table 4), a larger high-stress region forms (see Figure 15 specimen 0630). Original specimens prone to buckling did fail at these regions, as presented in our previous study [62]. Overall, localised high-stress regions are formed at the corners connected by the x-reinforcement, but no failure occurs during compression at any parameter combination. 

## 4. Discussion

Improving the mechanical performance of auxetic structures has attracted the interest of many fellow researchers. In our paper, we aimed to create a novel structure based on the doubly re-entrant structure whose deformation behavior is parameter-independent, making it predictable and easy to apply. Parameter-independent, non-buckling deformation behavior is achieved by the added x-reinforcement, while the mechanical properties are tunable based on geometrical parameters. Novel auxetic structures are generally created to improve mechanical properties. Fu et al. [50] in their research introduced a rhomboid-embedded re-entrant honeycomb structure and investigated the effect of geometrical parameters on mechanical properties. Research on improving the deformation behaviour of the re-entrant structure is limited, which is why we created our novel x-reinforced design. At the same time, fellow researchers are interested in improving the deformation behaviour of other auxetic structures; for example, Tomita et al. implemented a gradient design to control the buckling behaviour of origami-based auxetic structures [57]. 

Besides the achieved parameter-independent deformation behavior of our x-reinforced structure, its mechanical properties are greatly improved (up to four times greater energy absorption, a 406.2% increase) compared to the original doubly re-entrant structure. Owing to the deformation control effect of the x-reinforcement, the reinforced structures are no longer auxetic. It is important to note that re-entrant auxetic structures only show auxetic deformation at low deformation levels; re-entrant structures are prone to buckling, thus the auxeticity of this structure is inherently limited. The loss of auxeticity did not result in less favorable mechanical properties; as presented in our article, energy absorption and compression resistance is greater in the novel structure. In spite of that, an increased Poisson’s ratio results in reduced shear and fracture resistance [73], limiting the application of our structure for cyclic loads. 

Improving the mechanical or deformation properties of auxetic structures does not have to mean sacrificing auxeticity. Zhu et al. [48] aimed to overcome the conflict of auxetic performance by embedding elliptical structures. Mechanical properties (crushing stress) greatly improved, while auxeticity remained, although buckling occurred for some specimens. Further research results are discussed in Table 5. 

## 5. Conclusions

This paper presents a reinforced geometry modification. The goal is to make the doubly re-entrant lattice structure suitable for a wide range of industrial applications. Reinforcement is achieved with a nested geometry approach—the breakpoints of the unit cell are connected diagonally, which results in an “x” shape. To make a lattice structure suitable for a wide range of industrial applications, all of its properties must be known precisely and its behaviour must be parameter-independent. Furthermore, the task of the designer must be reduced to choosing the right parameters based on the provided design guidelines. The effect of the novel reinforced design was investigated on a broad parameter range. The x-reinforced design made the doubly re-entrant structure widely applicable, while its mechanical properties were as good or better than those of the original design. The following conclusions can be drawn from this study:

- The reinforced design improves the deformation stability of the doubly re-entrant lattice design, deformation behaviour is parameter-independent, and buckling does not occur;
- The reinforced, prominent centre unit cell segment results in stable, predictable behaviour; no sudden changes can be observed in any mechanical properties;
- The added x-reinforcement limits cell deformation; therefore, the lattice loses its auxetic behaviour, which, combined with localised stress regions, reduces the structure’s fracture resistance, limiting its application for cyclic loads;
- The novel structure has a low, nearly constant Poisson’s ratio even at a large deformation;
- In the case of x-reinforced geometries, decreasing the *deg* and *offset* parameters increases the compressive resistance in the investigated parameter range.

## Figures and Tables

**Table 1:** Parameter range to be investigated.

Specimen Name (X/ORIG)	0630_	0635_	0640_	1030_	1035_	1040_	1430_	1440_
$d_{0}$ (offset) [mm]	0.6	0.6	0.6	1.0	1.0	1.0	1.4	1.4
$\varphi_{0}$ (deg) [°]	30	35	40	30	35	40	30	35

**Table 2:** Print parameters.

Resin Print Parameters:	
Bottom layer exposure time:	40 s
Number of bottom layers:	6
Normal exposure time:	4 s
Layer thickness:	0.05 mm
Off time:	2 s
Z lift distance:	4.5 mm
Z lift speed:	0.5 mm/s
**Object Parameters:**	
Z lift height:	5 mm
Orientation relative to the build platform:	14 degrees
Support density:	Dense
Lowest anchor distance:	0.5 mm
Base plate thickness:	1 mm

**Table 3:** Material model properties of the unique resin mixture and the structural steel material.

Property	Value	Unit
**Tensile testing machine material: structural steel**		
Density	7850	$kg/m^{3}$
Ultimate tensile strength	460	MPa
Young’s modulus	200	GPa
Poisson’s ratio	0.3	[-]
**Specimen material: 75% Litliq FX 60 and 25% Litliq TH 50**		
Density	1.03	$kg/m^{3}$
Ultimate tensile strength at 5 mm/min	5.31	MPa
Material constant $C_{10}$	0.4850	MPa
Material constant $C_{01}$	1.8065	MPa
Incompressibility parameter	0.434782	1/GPa

**Table 4:** Deformation behaviour of specimens.

Parameter Designation	X-Reinforced Deformation Behaviour	Original (Doubly Re-Entrant) Deformation Behaviour
**0630**	Buckling	Lateral expansion
**0635**	Buckling	Lateral expansion
**0640**	Buckling	Lateral expansion
**1030**	Buckling	Lateral expansion
**1035**	Continuous auxetic	Lateral expansion
**1040**	Continuous auxetic	Lateral expansion
**1430**	Continuous auxetic	Lateral expansion
**1435**	Continuous auxetic	Lateral expansion
**1440**	Continuous auxetic	Lateral expansion

**Table 5:** Result discussion.

Structure	Result Evaluation	Reference
**Elliptical annular structure;** *based on the re-entrant honeycomb*	Improved mechanical properties: crushing strength, fracture resistance, slightly improved energy absorption (28%) Retained auxetic behaviour, with buckling	[48]
**Diagonally connected corner reinforcement**; *based on the re-entrant honeycomb*	Enhanced shear modulus, increased compression resistance (2.5 times) Initial auxetic deformation retained, but at large deformations buckling still occurs The diagonal reinforcement weakened the auxetic performance and significantly increased the Poisson’s ratio	[49]
**Self-similar inclusion;** *based on the re-entrant honeycomb*	Enhanced shear stress (up to 259%) and greatly improved energy absorption capability A more stable, controlled and self-collapsing, mostly auxetic, deformation At low velocity, uniform auxetic deformation is observed	[52]
**Embedded rhomboid; ** *based on the re-entrant honeycomb*	Increased stiffness even at low porosity, added stability Tunable mechanical properties based on clear instruction Varying Poisson’s ratio (negative and positive) Buckling occurs during deformation	[50]
**X-reinforced structure**; *based on the doubly re-entrant auxetic honeycomb.*	Increased deformation stability, specimens do not buckle Tuneable mechanical properties by varying mechanical properties Increased mechanical properties, up to 4 times greater energy absorption and up to 3 times greater compressive resistance The structure is no longer auxetic, characterised by low positive Poisson’s ratio	**Present** **design**

## Data Availability

The data presented in this study are available on request from the corresponding author.
